# Intravenous fish oil lipid emulsions in ICU patients: an updated systematic review and meta-analysis

**DOI:** 10.1186/cc14483

**Published:** 2015-03-16

**Authors:** P Langlois, R Dhaliwal, M Lemieux, D Heyland, W Manzanares

**Affiliations:** 1 Hospital Fleurimont, Sherbrooke, QC, Canada; 2 Kingston General Hospital, Kingston, ON, Canada; 3 Queen´s University, Kingston, ON, Canada; 4 University Hospital, Montevideo, Uruguay

## Introduction

Intravenous fish oil (FO) lipid emulsions (LEs) are rich in ω-3 polyunsaturated fatty acids, which exhibit anti-inflammatory and immunomodulatory effects. We previously demonstrated that FO-containing emulsions may be able to decrease mortality and ventilation days in the critically ill. Over the last year, several additional randomized controlled trials (RCTs) of FO-based LEs have been published. Therefore, the purpose of this meta-analysis was to update our systematic review aimed to elucidate the efficacy of FO-based LEs on clinical outcomes in the critically ill.

## Methods

We searched computerized databases from 1980 to 2014. Overall mortality was the primary outcome and secondary outcomes were infections, ICU and hospital length of stay (LOS), and mechanical ventilation (MV) days. We included RCTs conducted in critically ill adult patients that evaluated FO-based LEs in parenteral nutrition (PN) or enterally fed patients. We analyzed data using RevMan 5.1 (Cochrane IMS, Oxford, UK) with a random effects model.

## Results

**Figure 1:**
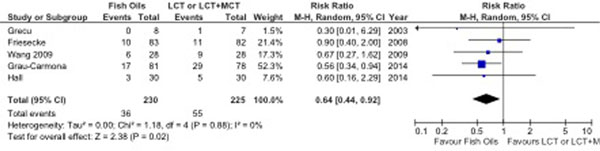
**Effects of parenteral fish oil lipid emulsions on infections**.

A total of 10 RCTs (*n *= 733), including four trials published over the last year, met inclusion criteria. There was considerable heterogeneity in interventions tested in these trials. No effect on overall mortality was found. When the Results of five RCTs that reported infections were aggregated, FO-containing emulsions significantly reduced infections (RR 0.64; 95% CI, 0.44 to 0.92; *P *= 0.02, heterogeneity *I*^2 ^= 0%). Furthermore, FO-based LEs were associated with a trend toward a reduction in MV days (WMD, -1.41; 95% CI, -3.43 to 0.61; *P *= 0.17, heterogeneity *I*^2 ^= 0%), and hospital LOS (WMD -4.06; 95% CI, -10.14 to 2.03; *P *= 0.19, *I*^2 ^= 89%, *P *< 0.00001), without effect on ICU LOS. See Figure 1.

## Conclusion

FO-based LEs may be associated with a reduction in infections, as well as clinically important reductions in duration of ventilation, and hospital LOS. Nevertheless, according to current literature there is inadequate evidence to give a final recommendation on the routine use of FO-containing emulsions in PN and/or as a pharmaconutrient strategy in enterally fed critically ill patients. Further large-scale RCTs which should aim to consolidate potential positive treatment effects are warranted.

